# A Rare Case of Idiopathic Painful Nervus Intermedius Neuropathy in a 13-Year-Old Female: A Case Report and Discussion in the Context of the Literature

**DOI:** 10.3390/children9081234

**Published:** 2022-08-15

**Authors:** Diego Fernandez-Vial, Linda Sangalli, Cristina Perez

**Affiliations:** Department of Oral Health Science, Division of Orofacial Pain, College of Dentistry, University of Kentucky, Lexington, KY 40536, USA

**Keywords:** earache, facial pain, gabapentin, geniculate neuralgia, nervus intermedius, neuralgia, neuropathic pain, otalgia

## Abstract

(1) Background: Painful nervus intermedius neuropathy involves continuous or near-continuous pain affecting the distribution of the intermedius nerve(s). The diagnosis of this entity is challenging, particularly when the clinical and demographic features do not resemble the typical presentation of this condition. To the best of our knowledge, only three case reports have described the occurrence of nervus intermedius neuropathy in young patients. (2) Case Description: A 13-year-old female referred to the orofacial pain clinic with a complaint of pain located deep in the right ear and mastoid area. The pain was described as constant, throbbing and dull, with an intensity of 7/10 on numerical rating scale, characterized by superimposed brief paroxysms of severe sharp pain. The past treatments included ineffective pharmacological and irreversible surgical approaches. After a comprehensive evaluation, a diagnosis of idiopathic painful nervus intermedius neuropathy was given, which was successfully managed with the use of gabapentin. (3) Conclusions and Practical Implications: The diagnosis and treatment of neuropathic pain affecting the nervus intermedius can be challenging due to the complex nature of the sensory innervation of the ear. The diagnosis can be even more challenging in cases of atypical clinical and demographic presentations, which in turn may result in unsuccessful, unnecessary, and irreversible treatments. Multidisciplinary teams and constant knowledge update are fundamental to provide good quality of care to our patients and not to overlook any relevant signs or symptoms.

## 1. Introduction

*The International Classification of Headache Disorders, 3rd edition* (ICHD-3), defines painful nervus intermedius neuropathy as pain within the distribution of the intermedius nerve, which innervates the auditory canal, auricle and region of the mastoid process [1]. It is usually described by the patient as a dull, deep pain felt in the ear, with a continuous or nearly continuous duration. Brief paroxysms may be superimposed, although not predominant as typical phenotype [1]. Additionally, sensory and/or mechanical stimuli applied to the posterior wall of the auditory canal accounts as common triggering factors [1].

The reports available in the literature interchangeably use the terms *nervus intermedius neuralgia* and *geniculate neuralgia* when referring to pain with neuropathic characteristics affecting the area innervated by the nervus intermedius. A recent review searching for articles published in PubMed before February 2020 on nervus intermedius neuralgia in the general population reported a total of 102 cases, with a mean age of 44.8 years old and a higher prevalence in females [2]. As for the etiology, the authors identified neurovascular conflict (48.0% cases, more frequently involving the anterior inferior communicating artery) and infections (10.8%, such as the Ramsay Hunt syndrome and chronic otitis media) as the most frequent causes, among other precipitating factors (30.3%, including masses, myelitis, arachnoid thickening or adhesions or iatrogenic causes). Conversely, 10.8% of the cases were classified as idiopathic [2]. 

The diagnosis of this entity is challenging, particularly when the clinical and demographic features do not resemble a typical presentation. To the best of our knowledge, only three case reports have described the occurrence of nervus intermedius/geniculate neuralgia in young patients [3,4,5], which supports its rare prevalence or high misdiagnosis. The aim of this report is to present a rare case of idiopathic painful nervus intermedius neuropathy in a 13-year-old female, originally misdiagnosed and unsuccessfully treated as otalgia secondary to ear infection. Only by supplementing the literature with atypical clinical presentations of this and other less prevalent conditions will it be possible to create awareness on challenging and often misdiagnosed orofacial pain conditions.

## 2. Case Presentation

A 13-year-old white female presented to the Orofacial Pain Clinic at the University of Kentucky (School of Dentistry, Lexington, USA), with a complaint of pain located inside the right ear and mastoid area. The patient indicated that the pain started 16 months prior, without an identifiable initiating factor. The pain was described as a constant throbbing and dull, with an intensity of 6–7/10 on a numeric rating scale (NRS, being 0 = no pain, and 10 = the worst possible pain, which has been validated in the pediatric population [6]). She also reported an average of 8–10 episodes of debilitating, sharp painful paroxysms per day, lasting for 1–2 minutes with an intensity of 9/10 NRS. The patient mentioned an average of one to two awakenings during the night, due to episodes of sharp pain. No trigger, aggravating or relieving factors were identified otherwise. 

Previous evaluations were performed by multiple specialists, including two otolaryngologists (ENT’s), a neurologist, her primary care physician, and a dentist. The patient underwent several imaging modalities with no pathological findings, which included a brain magnetic resonance image (MRI) with and without contrast, a head and neck computed tomography (CT), and panoramic and dental periapical radiographs. 

For a more comprehensive evaluation of the case, previous medical providers were contacted to review past diagnoses and treatments. It was determined that she was originally diagnosed with otalgia secondary to a possible ear infection (despite no signs of infection during the clinical examination). Thus, the patient was initially prescribed non-steroidal anti-inflammatory drugs (NSAIDs) and multiple trials of oral antibiotics, with no noticeable improvement. Subsequently, based on her history of previous ear infections, a tympanoplasty tube was surgically placed in the right ear, with no reported pain relief. 

The past medical history of the patient included asthma, seasonal allergies, and chronic migraine without aura. The patient specified that her headaches started when she was four years old, being located bilaterally in the forehead, with an intensity of 7–10/10 NRS, a frequency of 17 headache days per month, and a duration of 2–72 hours. Nausea and phonophobia were reported as associated factors, and the use of ibuprofen 800 mg and an external trigeminal nerve stimulation device as alleviating factors. She reported taking the following daily medications: topiramate 200 mg extended release, azelastine hydrochloride 0.15% nasal spray, levocetirizine dihydrochloride 5 mg, beclomethasone dipropionate 80 mcg inhaler, famotidine 20 mg, pseudoephedrine HCI 20 mg, fluticasone propionate 50 mcg spray, ibuprofen 800 mg (as needed, up to three times per week), and melatonin 3 mg. 

As for her psychosocial history, the patient was a full-time student and was alert, oriented and able to answer all the questions, not exhibiting any signs of confusion nor memory loss during the interview. She denied past and current use of tobacco, alcohol or any recreational drugs. She reported 9 hours of sleep, with a sleep latency of 5–10 minutes, and an average of two awakenings per night (sometimes because of pain). She denied symptoms of depression or anxiety, but manifested distress in relation to the continuous pain and the lack of a precise diagnosis. 

**Figure 1:**
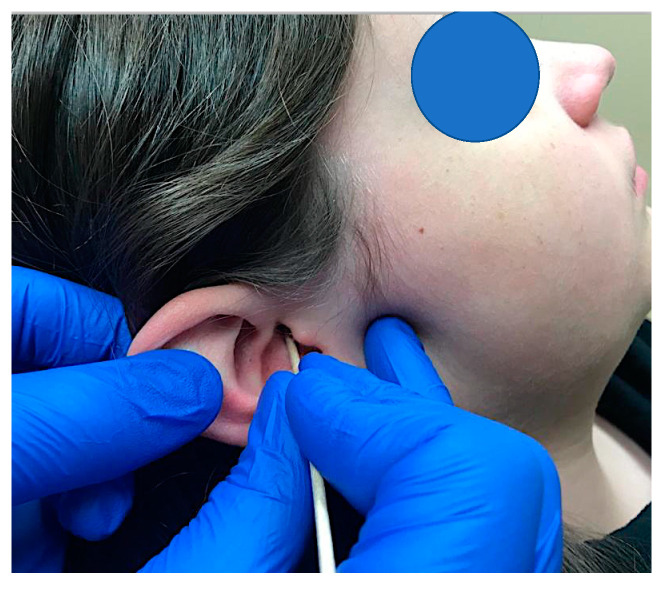
Stimulation of a trigger area in the posterior wall of the auditory canal with a cotton swab test (Q-tip test).

A comprehensive physical examination of the head and neck regions was performed. Cranial nerves II-XII were evaluated, with no sensory or motor deficits noted. A familiar shooting pain with an intensity of 9/10 NRS was elicited after cutaneous stimulation of a trigger area in the posterior wall of the auditory canal (Figure 1). The pain reportedly decreased after approximately 1 minute and returned to the constant throbbing and tender pain with an intensity of 6/10 NRS. The auditory canal was examined and the tympanoplasty tube did not show any sign of inflammation or infection. Head and neck movements were not painful nor restricted. Tenderness was elicited upon palpation of the right trapezius, right paracervical and right temporalis tendon. None of the forenamed muscles reproduced the patient’s chief complaint. To rule out referred pain originating from the temporalis tendon as a causative factor, a diagnostic anesthetic block was performed to the right temporalis tendon, which resulted in the resolution of the local tenderness in the area but did not produce any change in the ear and mastoid pain.

The medical provider that initially ordered the MRI (taken six months prior) was contacted to re-analyze the images with more emphasis on the cranial nerves V, VII, IX and X, and to rule out any intracranial pathology that may be responsible of the pain. The radiologist recommended a repeat of the study to analyze the specific segments related to the abovementioned nerves. A new brain MRI with and without contrast was obtained, which did not reveal any evident intracranial pathology, nor compression of cranial nerves or any other structures. 

After ruling out other pathologies associated with neuropathic pain affecting the cranial nerves V, VII, IX, and X, a diagnosis of *Idiopathic Painful Nervus Intermedius Neuropathy* (ICHD-3/13.3.2.4) was made.

## 3. Treatment/Management

In light of the diagnosis made and the potential interactions with the current daily medication regimen of the patient, a trial of 300 mg of gabapentin at bedtime was suggested. The patient was instructed to increase the initial 300 mg dose by adding one capsule every three days (one additional capsule AM, then PM, then at bedtime) depending on the evolution of her pain, and as long as no side effects were noticed. During the titration period, a constant communication was maintained between our team and the patient and her mother. After one month, at a dose of 2700 mg of gabapentin per day (900 mg three times per day), the patient reported a resolution of the constant throbbing pain, with residual episodes of sharp pain occurring three to four times per day, lasting for two minutes and with an intensity of 6–7/10 NRS. Additionally, she denied nocturnal awakenings due to pain. At a dosage of 3300 mg of gabapentin per day (1200 mg AM, 900 mg PM, and 1200 mg at bedtime), she reported the complete resolution of her complaint, and denied any side effects associated with the medication. Moreover, the patient reported a 40% reduction in migraine headache days per month, with a noticeable decrease in the intensity and duration. We discussed the case with the mother and decided to maintain this same dose for at least four to six months. 

After six months of not experiencing any episodes of pain in the ear or the mastoid area, and after discussing the case with the neurologist who was managing the patient’s migraine, we proceeded to attempt a decrease in the dose of gabapentin, at a rate of 300 mg every seven days. She was able to completely discontinue the medication for one month, after which she started experiencing similar episodes of shooting pain in the same area. Therefore, gabapentin was titrated up as previously done, and was successful in subsiding the pain again at a dose of 900 mg of gabapentin per day (300 mg three times a day). The patient remained pain free at the seven-month follow-up; gabapentin was maintained stable on the same dosage thereafter. The patient agreed to maintain regular follow-up appointments every six months, and the option of trying again to taper down the gabapentin will be reevaluated in the future if there is no reoccurrence of the pain.

## 4. Discussion

The nervus intermedius is a small branch of the facial nerve, and it contains parasympathetic and sensory fibers. When this nerve is affected by infections, impingement, compression (i.e., vascular compression or tumor), degeneration or other entities, symptoms of deep ear pain along with sporadic spikes of neuralgic or atypical pain may result [7,8]. The diagnosis and treatment of neuropathic pain affecting the nervus intermedius can be challenging, due to the complex nature of the sensory innervation of the ear, resulting in overlapping clinical features of other conditions. Therefore, a thorough differential diagnosis should include other neuralgias (e.g., neuralgia of the glossopharyngeal, trigeminal, and/or vagus nerves) or conditions affecting ears (e.g., red ear syndrome [9]), eyes, throat, nose, teeth, temporomandibular joints and other associated structures [10]. This diagnosis can become even more challenging in cases of atypical presentations, as described in the present report, which in turn may result in unsuccessful, unnecessary and sometimes irreversible treatments. 

Long-lasting undiagnosed pain conditions can increase the prevalence of associated psychological comorbidities, ranging from depression and anxiety to suicidal ideation [5]. This can, in turn, amplify pain perception and initiate and maintain the chronicity of the condition itself [11,12]. As such, an early diagnosis is extremely important, which may involve multiple specialists in an extensive workup, including neurologists, ENT’s, dentists, behavioral medicine and orofacial pain specialists.

Unfortunately, no established standard medical treatment exists for this condition, and most of the available options derive from treatments of other types of neuropathic pain in adults. From a review conducted on 114 cases of nervus intermedius neuralgia, the pharmacological management with carbamazepine was found to be the most common and effective approach (total of 30 cases; 18/30 good responders), followed by gabapentin (10 cases; 7/10 good responders) [2]. Both anticonvulsant drugs are widely used for the management of neuropathic pain. Specifically, carbamazepine and oxcarbazepine are considered as the first options for those neuropathic pain conditions that clinically present with paroxysmal episodes of pain (i.e., trigeminal and glossopharyngeal neuralgia) [13,14,15,16]; on the contrary, gabapentin is commonly used for continuous neuropathic pain presentations [17,18,19]. Other available systemic pharmacologic agents are pregabalin, serotonin and noradrenaline reuptake inhibitors (i.e., duloxetine and venlafaxine), tricyclic antidepressants (i.e., amitriptyline) and tramadol [13]. Second and third pharmacological lines of treatment include opioids, topical capsaicin, topical lidocaine and subcutaneous injection of onabotulinum toxin type A [13]. Unfortunately, none of the proposed medications have been specifically tested on the pediatric population with high-quality studies.

In case of the failure of a pharmacological approach, surgical interventions, including sectioning of the nervus intermedius, may be considered. This may be performed in conjunction with microvascular decompression of the nervus intermedius and of the adjunct cranial nerves IX and X [2,3,5,8]. Although those authors that support a surgical option describe a favorable outcome in 90% of the cases, other reports suggest that patients may develop atypical facial pain after an apparently successful surgical intervention [20]. Additionally, it has been reported that positive findings of compression or other nerve related pathologies in an MRI can be misleading and do not ultimately confirm the diagnosis of *Nervus Intermedius Neuropathy*; therefore, the contribution of imaging might not be considered highly sensitive and specific [5]. Due to the abovementioned considerations, a surgical approach should only be explored in refractory cases. In the present case, we opted for a trial with gabapentin as initial approach. Since pharmacological management resulted in a complete resolution of the pain and no abnormalities were found in the MRI, a surgical approach was not considered. 

### Case Discussion in the Context of Existing Literature

To the best of our knowledge, only three other case reports have been published on nervus intermedius neuralgia in the pediatric and adolescent population (Table 1). Another case report in the literature describes the diagnosis and successful resolution of intermedius neuralgia in a 1-year-old girl [21]. However, it has not been included in the present discussion as the condition was secondary to a brain schwannoma. 

All the reviewed cases reported in the literature share a severe pain intensity and an extensive list of unsuccessful trials, due to misdiagnosis of the conditions or to ineffective responses to medication. Interestingly, all the cases resolved with complete remission of the symptomatology at their longest follow-up (1 year in the study by Grin EJ et al. [4], 18 months in George DD et al. [5], and 5 years in Zenonos G et al. [3]). However, the three cases were managed with opposite treatments, *e.g.,* with antiepileptic and centrally acting medications in one case [4], with surgical intervention in the second case [5], and with a combination of surgery and anticonvulsants in the third case [3]. Despite all the cases resulting in complete remission of the symptomatology, there is a clear lack of a standardized treatment for this condition, especially in the pediatric population. Although studies with bigger sample size and randomized design are warranted, the occurrence of nervus intermedius neuralgia in youth is a rare finding. Thus, naturalistic studies and case reports may still be valuable with the aim of spreading knowledge and awareness among the clinicians. 

## 5. Conclusions

Nervus intermedius neuropathy is a rare condition, more so in young patients. Despite the persistent and painful manifestation, this type of neuropathic condition can be successfully managed with pharmacological modalities.

Constant update and understanding of the current literature, as well as multidisciplinary approaches, are fundamental to provide good quality of care to our patients. Signs and symptoms should not be overlooked or underestimated in order to obtain a proper diagnosis and, therefore, successful outcomes. It is important that practitioners recognize this condition and properly refer their patients to an appropriate provider, avoiding unnecessary and invasive procedures.

## Figures and Tables

**Table 1:** Nervus intermedius neuralgia in the literature.

Author and Study Characteristics	Pain Description (Location, Intensity, Quality, Pattern, Duration and Triggers)	Previous Treatments	Management and Outcomes at Last Follow-Up
Grin EJ et al. [4] - Case report - *n* = 1 (10 y.o.; male)	- Right ear, irradiating to right part of his throat and pre-auricular area - 10/10 sharp and stabbing, with 3/10 background pain; - Intermittent; - 5–10 s; - No triggers identified.	- Oral appliance; - Medications: gabapentin 3000 mg QD, hydrocodone-acetaminophen 5/325 mg PRN, oxycodone HCL 5 mg PRN, morphine sulfate 15 mg TID, antibiotics (cephalexin, cefdinir, sulfamethoxazole-trimethoprim, amoxicillin).	- Carbamazepine 200 mg TID + baclofen 10 mg BID + cognitive behavioural therapy + biofeedback + sertraline 50 mg QD; - 1-year follow-up: complete remission, no medications needed.
George DD et al. [5] - Case report - *n* = 1 (17 y.o.; male)	- Bilateral deep ear pain; - 9/10, stabbing; - Intermittent; - Triggers: touching the outer ear, wind and barometric changes.	- Medications: oxcarbazepine 300 mg BID, amitriptyline 50 mg QD, gabapentin 600 mg TID.	- Bilateral MVD of CNs VIII and IX + right CN X; sectioning of bilateral nervus intermedius; - 18-month follow-up: complete remission, no medications needed.
Zenonos G et al. [3] - Case report - *n* = 1 (9 y.o.; male)	- Right ear; - Intense sharp, “lightning bolt”; - Intermittent; - Few seconds; - Triggers: chewing and drinking.	- Medications: gabapentin 1500 mg QD, amitriptyline, and carbamazepine.	- Endoscopic MVD of CNs IX, X, and IX; sectioning of the NI + gabapentin 600 mg QD; - 5-year follow-up: complete remission.

QD: every day; PRN: as needed; TID: three times per day; BID: two times per day; MVD: microvascular decompression; CNs: cranial nerves; NI: nervus intermedius.

## Data Availability

Not applicable.
